# Exon array analysis reveals neuroblastoma tumors have distinct alternative splicing patterns according to stage and *MYCN *amplification status

**DOI:** 10.1186/1755-8794-4-35

**Published:** 2011-04-18

**Authors:** Xiang Guo, Qing-Rong Chen, Young K Song, Jun S Wei, Javed Khan

**Affiliations:** 1 Oncogenomics Section, Pediatric Oncology Branch, National Cancer Institute, National Institute of Health, Gaithersburg, MD 20877, USA; 2 Advanced Biomedical Computing Center, SAIC-Frederick, Inc., National Cancer Institute-Frederick, Frederick, MD 21702, USA

## Abstract

**Background:**

Neuroblastoma (NB) tumors are well known for their pronounced clinical and molecular heterogeneity. The global gene expression and DNA copy number alterations have been shown to have profound differences in tumors of low or high stage and those with or without *MYCN *amplification. RNA splicing is an important regulatory mechanism of gene expression, and differential RNA splicing may be associated with the clinical behavior of a tumor.

**Methods:**

In this study, we used exon array profiling to investigate global alternative splicing pattern of 47 neuroblastoma samples in stage 1 and stage 4 with normal or amplified *MYCN *copy number (stage 1-, 4- and 4+). The ratio of exon-level expression to gene-level expression was used to detect alternative splicing events, while the gene-level expression was applied to characterize whole gene expression change.

**Results:**

Principal component analysis (PCA) demonstrated distinct splicing pattern in three groups of samples. Pairwise comparison identified genes with splicing changes and/or whole gene expression changes in high stage tumors. In stage 4- compared with stage 1- tumors, alternatively spliced candidate genes had little overlap with genes showing whole gene expression changes, and most of them were involved in different biological processes. In contrast, a larger number of genes exhibited either exon-level splicing, gene-level expression or both changes in stage 4+ versus stage 1- tumors. Those biological processes involved in stage 4- tumors were disrupted to a greater extent by both splicing and transcription regulations in stage 4+ tumors.

**Conclusions:**

Our results demonstrated a significant role of alternative splicing in high stage neuroblastoma, and suggested a *MYCN*-associated splicing regulation pathway in stage 4+ tumors. The identification of differentially spliced genes and pathways in neuroblastoma tumors of different stages and molecular subtypes may be important to the understanding of cancer biology and the discovery of diagnostic markers or therapeutic targets in neuroblastoma.

## Background

Alternative splicing of pre-messenger RNA is nearly universal, involving more than 90% of human genes [1]. It is an important regulatory mechanism of gene expression for tissue-specific functions. Each gene maintains a delicate balance of its alternative transcripts in normal cells, disruption of which affects normal cellular processes and may cause various diseases, including cancer [2]. Although there have been numerous studies to identify tumor-specific splicing variants as diagnostic markers or therapeutic targets, only recently has alternative splicing in cancer been studied using genome-wide profiling methods [3-7]. Most of these studies focus on the identification of splicing variants in tumor, while little has been explored on the role of alternative splicing in tumors of different stages and molecular subtypes.

Neuroblastoma is the most common solid extracranial tumor in children. The incidence rate is 10.2 cases per million children under 15 years of age, and the median age at diagnosis is 17 months [8]. Stage, age, and other biological features in tumour cells are important prognostic factors for risk stratification and disease management. The "International Neuroblastoma Staging System" (INSS) classifies the tumor into six stages (1, 2A, 2B, 3, 4, 4S) according to its anatomical presence at diagnosis [9]. Localized disease has favorable outcome with an overall survival rate for stage 1 disease of 75-90%. Patients over 18 months with stage 4 disease has a 2-year disease-free survival rate of only 30-40% [10]. *MYCN *is the most important biologic marker for neuroblastoma. It is amplified in approximately 25% of de novo neuroblastoma cases and is more common in patients with advanced-stage disease. *MYCN*-amplified tumor is highly aggressive with poor outcome [11]. Alternative splicing has been shown to be involved in neuroblastoma development [12]. For example, kinesin family member 1B isoform beta (KIF1Bbeta) but not alpha is down-regulated in advanced stages of neuroblastoma. KIF1Bbeta induces apoptotic cell death, suggesting its role as a haploinsufficient tumor suppressor [13,14]. In this study, we used Affymetrix Human Exon 1.0 ST Array (HuEx) to measure exon expression levels in 47 neuroblastoma samples of different clinical stages and molecular subtypes including stage 1 with normal *MYCN *copy number (1-), stage 4 with *MYCN *amplification (4+) or normal *MYCN *copy number (4-). The goal was to identify stage- and *MYCN *amplification-specific splicing patterns in comparison to whole gene expression changes for the understanding of cancer biology and discovery of biomarkers or therapeutic targets in neuroblastoma.

## Results

### Identification of alternatively spliced candidate genes

**Figure 1:**
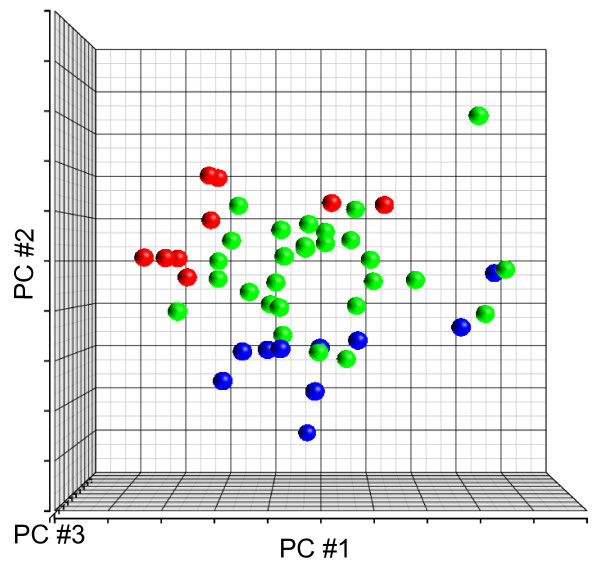
**Principal component analysis (PCA) of 47 neuroblastoma samples by log2-transformed normalized intensity of core probesets**. PCA was performed using NI values of core probesets (n = 221,809) across all samples after quality filtering. Stage 1- and 4+ samples are clearly separated from each other, while stage 4- samples are located between them. *Blue*, stage 1-; *green*, stage 4-; *red*, stage 4+ tumors.

To study the role of splicing regulation in high stage and *MYCN *amplified neuroblastoma, we used HuEx array to measure exon expression levels in 47 neuroblastoma samples from 10 stage 1-, 28 stage 4-, and 9 stage 4+ tumors (Table 1). HuEx array allows the detection of differential inclusion or skipping of exons by measuring expression levels of individual exons in different groups of samples. Alternative splicing events may be detected by normalized intensity (NI), which is defined as the ratio of exon-level probeset expression to gene-level transcript cluster expression [15]. To examine the global splicing pattern in neuroblastoma, we performed principal component analysis (PCA) using NI values of all core probesets (n = 221,809) across all samples after quality filtering. Figure 1 shows that stage 1- and 4+ samples are clearly separated from each other, while stage 4- samples are located between the other two groups. The separation of three groups of tumors by NI values suggests distinct alternative splicing patterns associated with clinical stage and *MYCN *status.

**Table 1:** Tumor samples used in the study

	Year of Diagnosis	Age at Diagnosis (years)	INSS Stage	*MYCN *Amplification Status	Clinical Outcome	Years of Survival
**NB7**	1998	1.3	1	NA	A	5.2

**NB8**	1998	4.6	4	NA	D	1.8

**NB9**	1996	1.1	1	NA	A	7.1

**NB17**	2000	1.2	1	NA	A	3.5

**NB21**	2000	5.2	4	AMP	D	0.6

**NB24**	2000	0.6	4	NA	A	3

**NB27**	2000	10.5	4	AMP	D	1.4

**NB29**	1998	0.3	1	NA	A	5.1

**NB30**	1997	0.9	4	NA	A	5.9

**NB31**	1998	1.4	4	NA	A	6.7

**NB32**	1998	1.2	4	NA	A	5.7

**NB33**	1998	1.4	1	NA	A	4.8

**NB34**	1997	1.2	1	NA	A	5.2

**NB205**	1995	3.9	4	NA	D	2.3

**NB206**	1995	2.7	4	NA	D	5.8

**NB207**	1995	4.4	4	NA	D	3.1

**NB208**	1995	0.8	1	NA	A	4.8

**NB210**	1996	2.5	4	NA	D	1.1

**NB221**	1997	0.4	1	NA	A	5.7

**NB237**	1999	4.0	1	NA	A	3.2

**NB238**	1999	1.1	1	NA	A	3

**NB265**	1996	1.8	4	AMP	D	2

**NB266**	1996	2.0	4	AMP	D	0

**NB269**	1997	0.8	4	NA	A	5.3

**NB275**	1995	1.6	4	NA	D	1

**NB278**	1999	1.7	4	AMP	D	0.8

**NB282**	1999	4.6	4	NA	A	3.3

**NB283**	1999	5.5	4	NA	D	4

**NB500**	1995	5.1	4	NA	A	7.4

**NB503**	2000	3.9	4	NA	A	3.3

**NB504**	2000	0.3	4	NA	A	3.5

**NB506**	2000	0.3	4	NA	A	3.9

**NB508**	2000	0.2	4	NA	A	3.2

**NB509**	2000	3.0	4	NA	D	4.1

**NB510**	2000	0.6	4	NA	A	3.4

**NB511**	2001	0.1	4	NA	A	3.0

**NB514**	2001	11.6	4	NA	A	3.5

**NB515**	2001	3.9	4	NA	A	3.2

**NB521**	1999	1.5	4	NA	A	5.6

**NB536**	1998	3.1	4	NA	D	1.2

**NB540**	1999	3.4	4	NA	D	1.1

**NB541**	1999	9.6	4	AMP	D	0.1

**NB543**	1999	11.4	4	NA	D	1.4

**NB545**	1999	3.4	4	AMP	D	2.2

**NB547**	2000	1.5	4	AMP	D	3.8

**NB548**	2000	1.6	4	NA	D	3.4

**NB581**	2002	1.6	4	AMP	D	1.6

Abbreviations: INSS, International Neuroblastoma Staging System; AMP, amplification; NA, not amplified; A, alive without event; D, deceased due to neuroblastoma disease.

Probesets with significantly different NI values in two groups of samples represent exons that may be differentially spliced in two disease states. We used Significant Analysis of Microarray (SAM) procedure [16] with a stringent false discovery rate threshold (q value < 0.05) to compare NI among three groups of patients. There were 1501 differentially spliced candidate genes between 4- and 4+, while only 362 genes were differentially spliced between 1- and 4-. Using AltAnalyze, we checked prior evidence of alternative splicing in Ensembl and/or UCSC genome browser databases [17]. Of the candidate gene lists derived from the comparison of 4-/4+ and 1-/4- tumors, 44.7% (671) of the former list and 29.6% (107) of the latter were supported by prior evidence of alternative splicing. The largest difference was between 4+ and 1- with 2775 differentially spliced candidate genes, of which 46.9% (1302) had alternative exons with prior evidence of alternative splicing (Additional file 1, Figure 2A). Therefore, both stage and *MYCN *amplification seem to affect the alternative splicing patterns in neuroblastoma.

**Figure 2:**
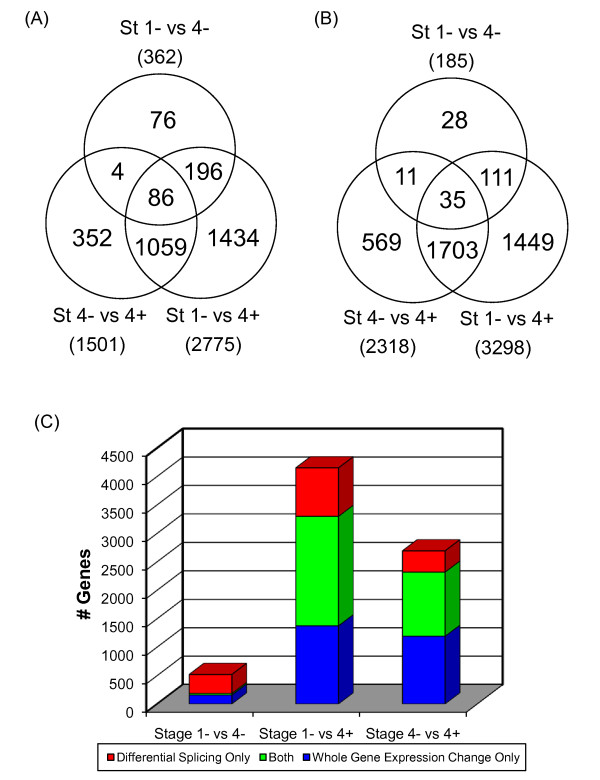
**Comparison of exon level splicing change and gene level expression change**. Pairwise comparison of stage 1-, 4-, and 4+ tumors was performed for both exon level splicing change and gene level expression change; the genes with FDR < 0.05 were identified. (A) Venn diagram of alternatively spliced candidate genes. (B) Venn diagram of differentially expressed candidate genes. (C) Comparison of exon level splicing change and gene level expression change. Blue column shows number of genes with whole gene expression change but not splicing change, while red column shows number of genes with splicing change but not whole gene expression change. Green column represents genes with both whole gene expression and splicing changes.

### Potential impact of alternative splicing in neuroblastoma

To estimate the functional impact of alternative splicing, we predicted changes in domain, motif, and miRNA binding site composition of protein sequences for alternatively regulated exons in stage 1-, 4-, and 4+ tumors [17]. The percentage of alternative exons that were associated with predicted domain/motif change ranges from 74.1% to 76.2% for the pair-wise comparison of three tumor groups. The number of genes including at least one alternative domain/motif were 296 (81.8%) for stage 4- vs 1-, 1181 (78.7%) for stage 4+ vs 4-, and 2203 (79.4%) for stage 4+ vs 1- respectively. In addition to the impact on protein domain/motif, alternative splicing may result in gain or loss of miRNA binding sites. Our analysis identified 19, 280, and 538 genes containing alternative exons overlapping with predicted miRNA binding sites for the comparison of 4-/1-, 4+/4-, and 4+/1- tumors, respectively. These results suggested diverse changes in protein function and expression regulated by alternative splicing in high stage neuroblastoma.

Several known aberrant splicing events in neuroblastoma and/or other tumors are evident in our candidate lists. For example, exon array data indicated decreased expression of KIF1Bbeta but not KIF1Balpha in high stage neuroblastoma, which is consistent with previous report (Additional file 2) [18].

**Figure 3:**
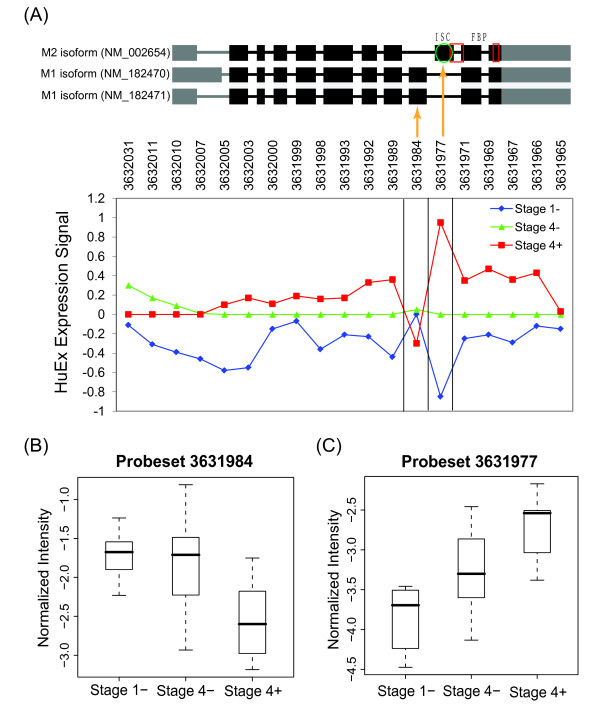
**Alternative splicing of pyruvate kinase (PKM2) detected by Affymetrix exon (HuEx) array**. (A) Gene structure of known isoforms is shown on the top panel with predicted domains/motifs that differ in protein isoforms. Green oval shows intersubunit contact (ISC) sequence, and red boxes point to fructose 1,6-bisphosphate (FBP) binding regions as defined by UniProt. The HuEx expression is shown on the bottom panel. Each point represents mean log2-expression of each group that was then median-centered across three groups. Orange lines point to probeset 3631984 that mapped to the unique exon in isoform M1 (NM_182470 and NM_182471), and probeset 3631977 that mapped to the unique exon in isoform M2 (NM_002654). While isoform M2-specific probeset showed increased expression in stage 4+ compared to stage 4-/1- tumors, isoform M1-specific probeset indicated lower expression in stage 4+ tumor. (B) Normalized intensity (NI) values for probeset 3631984 in Stage 1-, 4- and 4+ tumors. (C) Normalized intensity values for probeset 3631977 in Stage 1-, 4- and 4+ tumors. The expression for probesets 3631984 and 3631977was significantly different between stage 1- and 4+, suggesting the increased expression of isoform M2 and reduced expression of isoform M1 in *MYCN*-amplified neuroblastoma.

More interestingly, our results showed increased expression of M2 isoform of PKM2 in high stage neuroblastoma while the M1 isoform exhibited decreased expression (Figure 3). The splicing switch of pyruvate kinase has been demonstrated in multiple tumor types [19], but not previously reported in neuroblastoma. Two isoforms are expressed through exchange of two cassette exons, which are equally long and share 60% protein sequence identity [20]. Functional annotation by AltAnalyze identified the gain of the allosteric activator fructose 1,6-bisphosphate (FBP) binding region and intersubunit contact in high stage neuroblastoma, consistent with the allosteric regulation of M2 isoform but not M1 isoform by FBP [20]. The upregulation of M2 isoform in stage 4+ vs 1- tumors was also evident at the proteome level demonstrated by a previous proteomics study in our lab, which applied isotope-coded affinity tags (ICAT) in combination with mass spectrometry to quantify peptide expression levels in stage 1- and 4+ tumors [21]. The quantitative ICAT analysis identified five peptides matched against PKM2 protein sequence, among which KCCSGAIIVLTKS was from the exon specific to the M2 isoform and the other four were from constitutive exons in PKM2. M2 isoform-specific peptide demonstrated a mean log2-transformed expression ratio of 2.73, and the other peptides had a mean ratio of 1.67 between 4+ and 1- tumors ([21]). Although no peptide has been found for M1 isoform-specific exon, the higher expression change of M2 isoform-specific exon than constitutive exons suggested the upregulation of M2 isoform but not the M1 isoform at the proteome level.

### Alternative splicing and whole gene expression changes in neuroblastoma

To compare global splicing and transcription regulation in high stage neuroblastoma, we derived whole gene expression signatures by pairwise comparison of gene-level transcript cluster signals. Therefore, splicing signature includes genes having differentially included/excluded exons between two tumor groups, while whole gene expression signature are genes with different gene-level signals of two tumor groups. SAM analysis resulted in 185, 2318, and 3298 genes showing whole gene expression changes for stage 1- vs 4-, 4- vs 4+, and 1- vs 4+, respectively (FDR ≤ 0.05 and Fold Change ≥ 1.5; Figure 2B, Additional file 3).

**Figure 4:**
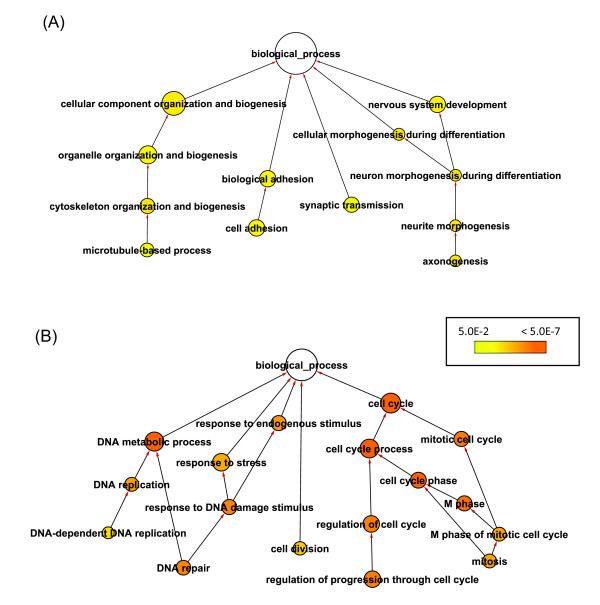
**Enriched Gene Ontology (GO) biological processes in alternatively spliced (A) or differentially expressed (B) genes in stage 4- vs 1- neuroblastomas**. GO enrichment analysis was done by DAVID [22], and overrepresented biological processes were shown as a GO graph in which child terms are connected to their parent terms by directed lines. Color scale denotes Benjamini-corrected p-values generated by a hypergeometric test, and node size is proportional to the number of genes annotated to corresponding GO terms.

Comparing stage 4- with 1-, only 27 genes were shared between splicing signature (n = 362) and whole gene expression signature (n = 185) (Figure 2C). Using the Database for Annotation, Visualization and Integrated Discovery (DAVID) [22], we found that genes with known splice variants were significantly overrepresented in splicing signature (n = 153) but not expression signature based on UniProt annotation (FDR < 0.05), which indicated the validity of our splicing analysis process. The most enriched Gene Ontology terms in the biological process category in the splicing signature included nervous system development, cell adhesion, synaptic transmission, and cytoskeleton organization and biogenesis (Figure 4A, Additional file 4). In contrast, the whole gene expression signature is enriched with genes involved in cell cycle, cell division, and DNA metabolic processes (Figure 4B, Additional file 4), which is consistent with the results derived using traditional gene expression microarray platforms [23]. Different sets of genes with different biological functions were affected by alternative splicing and transcription regulation, suggesting independent roles of splicing and transcription regulation in stage 4- neuroblastoma.

*MYCN *amplified neuroblastoma is the most aggressive disease subtype. By comparing stage 1- and stage 4+ neuroblastomas, we identified 9044 probesets within 2775 alternatively spliced candidate genes, along with 3298 differentially expressed genes between two tumor groups. Again, significant enrichment of genes with known splice variants (n = 311) was found in those showing only splicing changes (n = 852), but not those having only whole gene expression changes (n = 1375) (FDR < 0.05). In contrast to stage 4- tumors, a large number of genes (n = 1923) appeared to have both changes in stage 4+ tumors (Figure 2C). Biological processes enriched in alternatively spliced genes in stage 4+ include those involved in stage-specific splicing signature and whole gene expression signature (Table 2). Similar GO terms were enriched in genes showing whole gene expression changes in stage 4+ (Additional file 5). To derive *MYCN *amplification-specific signatures, we compared stage 4- and 4+ tumor, and detected 4602 probesets within 1501 genes that may undergo splicing disruption, and 2318 transcript clusters with expression changes during *MYCN *status change. The overlap between two signatures was also large with 1127 genes in common (Figure 2C). GO enrichment analysis results were similar to what was found in the comparison between stage 4+ vs 1- tumors (Additional file 6). These results suggested a correlation between *MYCN *amplification and splicing regulation. While splicing and transcription regulation may affect different sets of genes involved in different biological processes in stage 4- tumors, these biological processes may be disrupted by both gene expression regulatory processes to a greater extent in the more severe stage 4+ neuroblastoma.

**Table 2:** Top 40 overrepresented Gene Ontology biological processes (FDR < 0.0001) in alternatively spliced genes in stage 4+ vs. 1- tumors

GO Term	# Gene	PValue
cell cycle process **	203	2.26E-15

mitotic cell cycle **	107	8.64E-15

cellular component organization and biogenesis *	572	1.07E-14

cell cycle **	230	1.33E-14

cell cycle phase **	115	2.03E-14

mitosis **	84	2.29E-14

cell division **	88	3.86E-14

M phase of mitotic cell cycle **	84	4.03E-14

M phase **	97	1.28E-13

organelle organization and biogenesis *	285	1.67E-13

developmental process	651	4.37E-12

nervous system development *	192	1.66E-10

multicellular organismal development	479	4.24E-10

cellular process	2167	4.71E-10

chromosome segregation	32	7.47E-10

anatomical structure development	441	1.43E-9

localization	610	1.10E-8

regulation of cell cycle **	135	1.11E-8

cell cycle checkpoint	30	1.15E-8

cytoskeleton organization and biogenesis *	134	1.41E-8

system development	364	1.93E-8

regulation of progression through cell cycle **	133	2.61E-8

DNA replication **	74	7.81E-8

synaptic transmission *	84	2.63E-7

transmission of nerve pulse	93	2.98E-7

establishment of localization	533	5.36E-7

intracellular signaling cascade	307	6.77E-7

cell proliferation	179	7.05E-7

transport	516	7.30E-7

mitotic sister chromatid segregation	18	7.33E-7

response to DNA damage stimulus **	86	1.18E-6

sister chromatid segregation	18	1.33E-6

biological adhesion *	173	1.67E-6

cell adhesion *	173	1.67E-6

DNA metabolic process **	188	2.59E-6

cell communication	747	7.35E-6

interphase	34	1.15E-5

DNA replication initiation	16	2.45E-5

DNA-dependent DNA replication **	36	2.54E-5

cell differentiation	355	2.55E-5

* GO terms that are also enriched in alternatively spliced genes in stage 4- vs 1- tumors.

** GO terms that are also enriched in differentially expressed genes in stage 4- vs 1- tumors.

### Validation of splice variants differentially expressed in stage 1- and stage 4+ tumors

**Figure 5:**
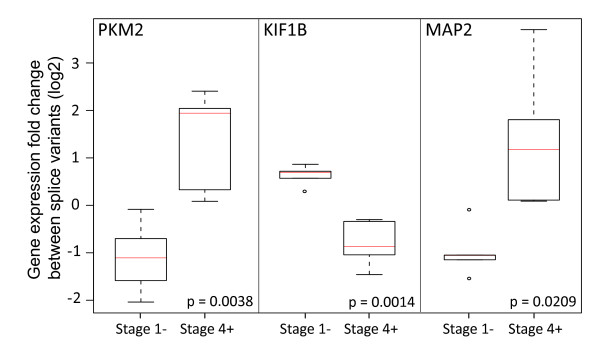
**qRT-PCR validation on splice variants differentialy expressed in stage 1- and stage 4+ tumors**. Quantitative RT-PCR was performed for three spliced genes including PKM2 (NM_002654 vs. NM_182470 & NM_182471), KIF1B (NM_015074 vs. NM_183416) and MAP2 (NM_001039538 vs. NM_002374) in 5 stage 1- and 5 stage 4+ tumors. Differential expression of splice variants was evaluated by calculating expression fold changes between splice variants of the spliced gene in each sample, which were further centered by median of values obtained in stage 1- and stage 4+ tumors. The expression of splice variants is significantly different between stage 1- and stage 4+ tumors for all three tested spliced genes.

Three alternatively spliced genes have been selected for quantitative RT-PCR validation including PKM2 (NM_002654 vs. NM_182470 & NM_182471), KIF1B (NM_015074 vs. NM_183416) and MAP2 (NM_001039538 vs. NM_002374). Figure 5 shows gene expression fold changes between splice variants in 5 stage 1- and 5 stage 4+ tumors. The HuEx results for all three alternatively spliced genes were validated by qRT-PCR result and the expression of splice variants is significantly different between stage 1- and stage 4+ tumors with p-values (t-test) equal to 0.0038, 0.0014 and 0.0209 for genes PKM2, KIF1B and MAP2 respectively. The expression of M2 isoform (NM_002654) of PKM2 is increased in stage 4+ compared with M1 isoform; the expression of KIF1Bβ (NM_015074) is decreased in stage 4+ compared with KIF1Bα (NM_002374); and the expression of isoform NM_001039538 of MAP2 is higher in stage 4+ compared with the isoform NM_002374.

## Discussion

Alternative splicing of precursor mRNA is an essential step in gene expression and responsible for much of the proteome diversity in mammalian genomes. Although splice variants have long been known to be associated with many human diseases, very little is understood about the global properties of alternative splicing in cancer development. Using Affymetrix Human 1.0 exon array, we compared splicing interruption and whole gene expression change in different stages of a pediatric cancer - neuroblastoma. Our results suggested a significant role of splicing regulation in high stage and *MYCN *amplified neuroblastoma tumors.

In stage 4- neuroblastoma, transcription regulation and alternative splicing may affect different sets of genes involved in different biological processes with more genes showing splicing disruption than whole gene expression change. Regulation of transcription and splicing seem to be two independent processes that result in distinct functional outcomes in stage 4- tumor, which is consistent with the independent roles of these two processes in determination of tissue specificity [24] and regulation of immune response [25]. In contrast, a large group of genes underwent both splicing and whole gene expression changes in stage 4+ tumor. Similar biological processes were enriched in splicing and whole gene expression signatures in stage 4+ tumor, including those that were affected separately by alternative splicing and whole gene expression changes in stage 4- tumor. It indicates that different biological processes may be affected by splicing and transcription regulation in stage 4- tumors and these processes may need to be disrupted by both gene regulatory processes in the more severe stage 4+ tumors.

*MYCN-*amplified tumor is a highly aggressive subtype with poor prognosis in 20% of neuroblastoma patients. Several studies have shown the differential expression of a large number of genes involved in cell cycle and differentiation in these tumors [23,26]. Our current study indicates, for the first time, that *MYCN *amplification is not only related to large scale gene expression changes but also profound splicing regulation in neuroblastoma. *MYCN *is a global transcriptional regulator for both protein-coding genes and genes encoding non-coding RNA products [27,28]. Transcriptional targets for *MYCN *include splicing factors [29] which may in turn regulate alternative splicing of various target genes. Recent study has shown that the splicing switch of pyruvate kinase in human gliomas may be controlled by c-myc through splicing factors including polypyrimidine tract binding protein (PTBP1) [30]. The PKM2 splicing switch (Figure 3) and PTBP1 expression upregulation we found in stage 4+ neuroblastoma (Additional file 3) suggest a *MYCN*-controlled pathway for PKM2 splicing in neuroblastoma. Other splicing targets of PTBP1 have also been found in our splicing signatures of *MYCN*-amplified tumors, including reticulon 4 (RTN4) [31] and ROD1 regulator of differentiation 1 [32]. In addition, multiple differentially expressed *MYC/MYCN *target genes in stage 4+ tumor appear to be associated with splicing processing complex, such as small nuclear ribonucleoprotein polypeptides (SNRPA, SNRPB, SNRPD2), dead-box polypeptides (DDX1, DDX18), RNA-binding motif protein (RBM3), and cleavage and polyadenylation specific factor (CPSF1) [29,33]. These splicing regulators may be involved in *MYCN*-associated splicing regulation, through which *MYCN *may exert part of its phenotypic effects on neuroblastoma.

Alternative splicing plays important roles in various diseases. It may be a direct cause of the disease, or a modifier of disease susceptibility and severity [2]. In this study, we identified candidate genes undergoing splicing disruption in high stage and *MYCN *amplified neuroblastoma, which help the understanding of disease biology. Neural development and cell adhesion genes exhibit splicing changes in stage 4- disease, and they undergo both splicing regulation and whole gene expression level change in stage 4+ patients. Previous proteomics study has demonstrated that proteins involved in these processes are significantly suppressed in stage 4+ neuroblastoma patients [21]. Our results suggest that alternative splicing may be responsible, at least partially, for the changes observed at the proteome level. Defects in splicing machinery may cause alternative splicing and further whole gene expression changes of neural development and adhesion genes, resulting in the protein level changes observed in our previous study.

Alternatively spliced candidate genes identified in this study provides a useful resource for the discovery of diagnostic biomarkers or therapeutic targets in neuroblastoma. One example is related to splicing switch of PKM2 variants. Previous studies in multiple tumor types have shown that M2 isoform is expressed while M1 isoform disappears during tumor development [34]. PKM2 plays important role in cancer metabolism, and it has been proposed to be a potential metabolic target for the treatment of cancer [19,35]. Our results demonstrated the splicing switch of isoform M1 to isoform M2 in high stage neuroblastoma (Figure 3), suggesting a similar role of PKM2 in neuroblastoma development as in other tumors [35]. Another interesting result is related to the alternative splicing of microtubule associated genes. Our data demonstrated that genes involved in microtubule-associated process were significantly enriched in differentially spliced candidate genes in stage 4+/4- vs stage 1- tumors. There were 57, 27, and 15 microtubule-associated genes showing aberrant splicing pattern in stage 4+ vs 1-, 4+ vs 4-, and 4- vs 1- respectively (Figure 4A; Additional file 4, 5, 6). Among these genes, KIF1Bbeta but not alpha has been shown to be a potential 1p36 tumor suppressor for neuroblastoma. Protein regulator of cytokinesis 1 (PRC1) is essential for organization of central spindle and midzone formation, whose interaction with KIF2C has been shown to be involved in breast cancer tumorigenesis [36]. Microtubule-associated protein 2 (MAP2) has been shown to be a prognostic marker for melanoma patients [37], and splice variants of MAPT gene demonstrated opposite changes in normal versus prostate tumor [38]. Recent studies have suggested a significant role of deregulated microtubule dynamics in enhanced genomic instabilities and tumor development [39]. Our results suggest that alteration of microtubule dynamics by alternative splicing may be an important pathogenetic mechanism in high stage neuroblastoma. Further functional study of these microtubule-associated genes may reveal novel tumor suppressors or oncogenes for neuroblastoma.

## Conclusions

In summary, our study demonstrated the important roles of splicing regulation in high-stage and *MYCN *amplified neuroblastoma. There may be a generalized shift in global splicing patterns synchronizing with the development of malignant phenotypes of tumor cells. Deciphering the "splicing code" is essential for our understanding of cancer etiology and progression pathway.

## Methods

### Tumor samples

We used 47 pretreatment primary neuroblastoma tumor samples in our study. All patients were blinded and anonymized to us. Our protocol was exempt from the NIH Multiple Project Assurance and our research activity involving human subjects was exempt from the office of Human Subjects Research (OHSR). The median age of forty-seven patients at diagnosis was 1.6 years (range 0.1 - 11.6 years). Ten patients had stage 1 disease while the remaining thirty-seven patients had stage 4 disease of which nine demonstrated *MYCN *gene amplification.

### Array experiments and data analysis

We extracted total RNA from tumor samples [40], which were then processed and labeled using Affymetrix whole transcript one-cycle labeling kit. After hybridization to Affymetrix Human Exon 1.0 arrays, chips were stained and scanned as per manufacturer's instructions. For data analysis, we used Affymetrix Power Tool (APT) to get signal intensities for probesets and transcript clusters. Probeset signals were estimated by PLIER and a detection p-value was assigned to each probeset by DABG algorithm. Gene-level signals were derived for 17800 transcript clusters comprised of core probesets that are supported by RefSeq transcripts and full-length mRNAs using IterPLIER algorithm. IterPLIER identifies highly correlated probesets of each transcript cluster to derive transcript cluster signal, thus gene-level expression estimate mostly includes the expression level of constitutive exons, which reflects gene expression regulation at the level of transcription and/or RNA stability but not splicing. A probeset is considered to be expressed in a sample if its detection p-value is less than 0.05, and a transcript cluster is expressed if more than half of its consisting probesets have p values less than 0.05.

For each core probeset (~284,000), we calculated normalized intensity (NI) which is probeset (exon-level) intensity divided by transcript cluster (gene-level) intensity in each sample. The comparison of NI between two sample groups may reveal exons that are differentially spliced in two groups. To reduce the false positive rate in the splicing variant identification, several filtering steps were applied to the signals of both probesets and transcript clusters. First, the detection *p*-value was used to remove probesets and transcript clusters with undetectable signals. Probesets are required to be expressed in >50% samples of at least one group, while transcript clusters have to be expressed in >50% samples of both sample groups. Next, we removed probesets with very low variances based on the interquantile range of probeset intensities across all samples. The least variable 20% probesets were discarded to reduce the number of cross-hybridizing or non-responsive probesets. Thirdly, we set expression values of less than 30 to 30 to reduce noise [7]. Lastly, we reduced the effect of inaccurate annotations by removing transcript clusters with more than one annotated Entrez Gene identifiers as well as multiple transcript clusters annotated by the same Entrez Gene identifier.

After the filtering steps, we used the Significance Analysis of Microarray (SAM) [16] two class unpaired method to identify probesets that have statistically significant changes in NI values between two groups of patients. Briefly, a score *d*_*i *_is calculated for each probeset to measure the relative difference in splicing index between two groups of samples,

where *s*_*i *_is a pooled standard deviation over the two groups of samples, and s_o _is a small positive constant that adjusts for the small variability in the data. These *d*_*i *_values are used to rank probesets on ascending order and derive observed order statistics *d(i)*. Then, a permutation procedure (n = 100) is applied to get a set of permuted relative difference values and corresponding order statistics for each probeset. The average of permuted order statistics is defined as the expected statistic *d*_*E*_*(i)*, which is plotted versus the observed statistic *d(i) *in a scatter plot. The set of probesets that are away from the *d(i) = dE(i) *line by a distance greater than an adjustable threshold Δ are called significant, and the percentage of such probesets identified by chance (false discovery rate) is estimated by the permuted dataset. In our study, the threshold Δ was chose to achieve a false discovery rate of 0.05, and the fold change of NI was required to be more than 1.5. The analysis of SAM was done by the R package *samr*. Similarly, differentially expressed genes were identified by applying SAM on the signal estimate of transcript clusters. Non-specific filtering was applied to remove transcript clusters that are only expressed in less than 20% of all samples. The false discovery rate is also required to be less than 0.05, and the signal fold change is more than 1.5.

For each probeset with significantly different NI between two tumor groups, we used AltAnalyze to identify competitive transcript isoforms that contain or do not contain the exon overlapping with that probeset. Possible splicing events caused by the alternative exon were classified into seven types (alternative N/C-terminus, alternative cassette exon, alternative 5'/3' splice site, retained intron, bleeding exon) based on the comparison of exon structures of competitive isoforms. Furthermore, we predicted changes in protein domains/motifs and miRNA binding sites associated with the probeset using both competitive isoform analysis and direct alignment method in AltAnalyze [17,41].

Gene-level signal was estimated by the IterPlier algorithm, and it mostly includes the expression level of constitutive exons, which reflects gene expression regulation at the level of transcription and/or RNA stability but not splicing.

### Array data access and visualization

To facilitate exon array data access and visualization, we have developed a novel database and visualization system displaying both gene-level and exon-level expressions for array probesets and transcript clusters (http://pob.abcc.ncifcrf.gov/cgi-bin/JK). Coupled with exon array annotation tools, such as ArrayCheck [42], this system allows easy query and visual inspection of alternatively spliced exons. Clinical outcome was also integrated with gene expression data such that Kaplan-Maier plot may be dynamically generated for each probeset and transcript cluster. The raw data and the processed data are also available at http://www.ncbi.nlm.nih.gov/geo/ (GEO accession: GSE27608).

### Quantitative RT-PCR validation

One microgram of total RNA was reversely transcribed to cDNA using Superscript II reverse transcriptase (Invitrogen) and random hexamer primer (Invitrogen) at 42°C for 1 hour. PCR reactions were performed with 40 cycles at 95°C for 30 sec, 60°C for 30 sec, and 72°C for 45 sec using SYBR Green PCR Master Mix (Applied Biosystems) and spicing variant-specific primers on ABI 7000 Sequence Detection System (Applied Biosystems). Splice variant-specific primers were designed using Primer 3 program (http://frodo.wi.mit.edu/primer3) and synthesized by Integrated DNA Technologies. Primer sequences are as follows: PKM2 (NM_182470&NM_182471) forward 5'-CTA TCC TCT GGA GGC TGT GC-3', reverse 5'-GAG GCT CGC ACA AGT TCT TC-3'; PKM2 (NM_002654) forward 5'-ATC GTC CTC ACC AAG TCT GG-3', reverse 5'-GAA GAT GCC ACG GTA CAG GT-3'; KIF1Bα (NM_183416) forward 5'- GAA GAT CGA AGA CGT CAT GGC C-3', reverse 5'-ACA CCA GCA CCA ACA GGC TCC-3'; KIF1Bβ (NM_015074) forward 5'-ACT TCT AGC TGG CAC AAT ACG-3', reverse 5'-GTC ACC GTC AAG AAT CAC AAA G-3'; MAP2 (NM_002374) forward 5'-TGG GTG GAC ACT CAA GAT GA-3', reverse 5'-TGA TCT CCG AGC TTC CTT TT-3'; MAP2 (NM_001039538) forward 5'-GCT CTG GCT CCC AGT GTA TT-3', reverse 5'-CTG CTG AGG TGG GCT GTA TT-3'.

To evaluate differential expression of splice variants in stage 1- and stage 4+ tumors, we calculated expression fold changes between different splice variants of the spliced gene in each sample, which were further centered by median of values obtained in stage 1- and stage 4+ tumors.

## Competing interests

The authors declare that they have no competing interests.

## Authors' contributions

XG and QRC performed data analysis and wrote the manuscript. YKS carried out the experiments and helped interpret the data. JSW helped data analysis and revised the manuscript. JK conceived and supervised the study, and revised the manuscript. All authors read and approved the final manuscript.

## Pre-publication history

The pre-publication history for this paper can be accessed here:

http://www.biomedcentral.com/1755-8794/4/35/prepub

## Supplementary Material

Additional file 1 **Alternatively spliced candidate genes among stage 1-, 4-, and 4+ tumors detected by HuEx exon array study (FDR < 0.05)**. Click here for file

Additional file 2 **Alternative splicing of kinesin family member 1B (KIF1B) detected by Affymetrix exon array**. While KIF1Balpha-specific probesets (6 probesets labeled by orange line) had no significant expression change, KIF1Bbeta-specific probesets (31 probesets to the right of the orange line) had significantly lower expression in high stage tumors. Click here for file

Additional file 3 **Candidate genes with whole gene expression changes among stage 1-, 4-, and 4+ tumors detected by HuEx exon array study (FDR < 0.05)**. Click here for file

Additional file 4 **Gene Ontology enrichment of alternative spliced and/or differentially expressed candidate genes in stage 4- vs 1- tumors**. Click here for file

Additional file 5 **Gene Ontology enrichment of alternative spliced and/or differentially expressed candidate genes in stage 4+ vs 1- tumors**. Click here for file

Additional file 6 **Gene Ontology enrichment of alternative spliced and/or differentially expressed candidate genes in stage 4+ vs 4- tumors**. Click here for file
