# Supplementary figures and images for: Exon array analysis reveals neuroblastoma tumors have distinct alternative splicing patterns according to stage and MYCN amplification status
Source: BMC Med Genomics. 2011 Apr 18;4:35. doi: 10.1186/1755-8794-4-35 (PMC3096898; doi:10.1186/1755-8794-4-35)

# Additional File 2

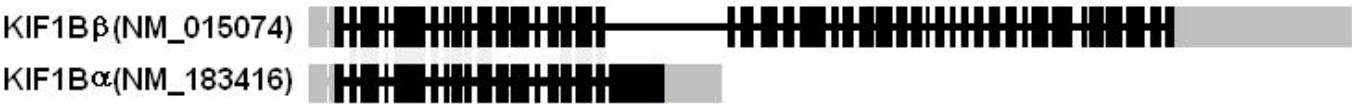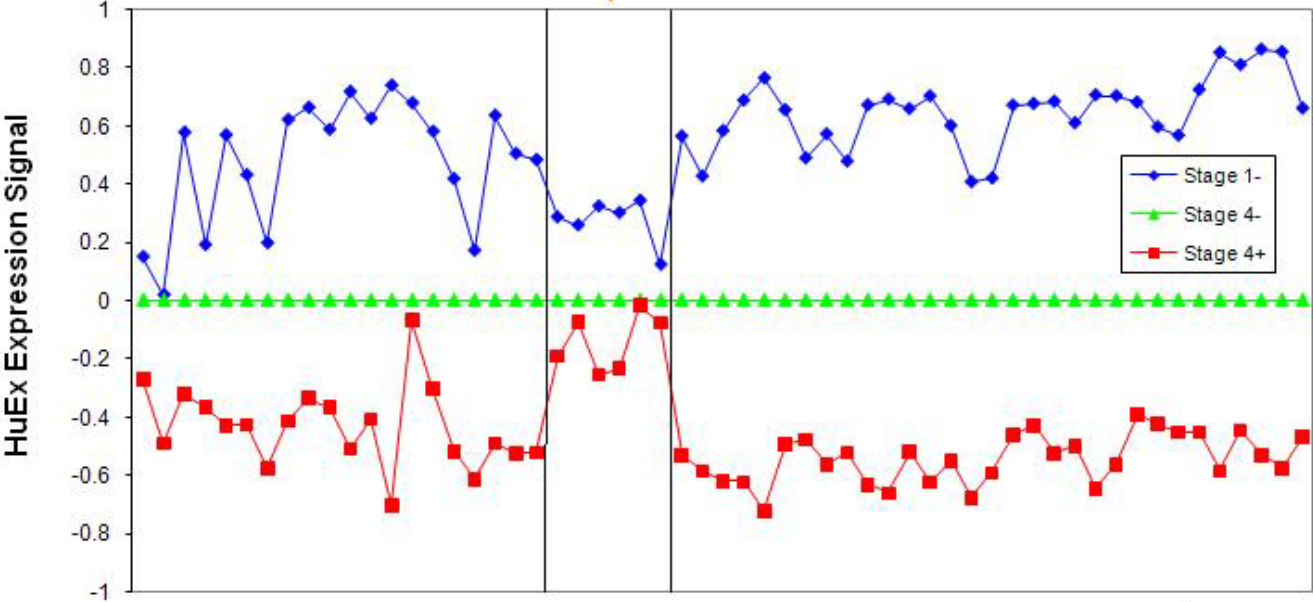

Supplement: Additional file 2 — Alternative splicing of kinesin family member 1B (KIF1B) detected by Affymetrix exon array. While KIF1Balpha-specific probesets (6 probesets labeled by orange line) had no significant expression change, KIF1Bbeta-specific probesets (31 probesets to the right of the orange line) had significantly lower expression in high stage tumors. [file 1755-8794-4-35-S2.PDF]
